# OSCAR facilitates malignancy with enhanced metastasis correlating to inhibitory immune microenvironment in multiple cancer types

**DOI:** 10.7150/jca.51964

**Published:** 2021-05-05

**Authors:** Xia Liao, Yang Bu, Yaoyao Zhang, Binghui Xu, Junrong Liang, Qingan Jia, Chun Zhang

**Affiliations:** 1 Department of Nutrition, First Affiliated Hospital of Xi'an Jiaotong University, Xi'an 710061, China.; 2 Department of Hepatobiliary Surgery, People' hospital of Ningxia Hui Autonomous Region, Yinchuan 750001, China.; 3 Institute of Medical Research, Northwestern Polytechnical University, Xi'an 710072, China.; 4 State Key Laboratory of Cancer Biology, National Clinical Research Center for Digestive Diseases and Xijing Hospital of Digestive Diseases, Fourth Military Medical University, Xi'an 710032, China.; 5 Department of Hepatobiliary Surgery, First Affiliated Hospital of Xi'an Jiaotong University, Xi'an 710061, China.

**Keywords:** OSCAR, immune microenvironment, macrophage, metastasis, prognosis

## Abstract

Cross talk between tumors and the immune microenvironment play a critical role in the malignant progression. The osteoclast-associated receptor (OSCAR) is a regulator of lymphocyte differentiation and maturation, but little is known about the role of OSCAR in multiple cancer types. We comprehensively analyzed OSCAR expression and explored its correlation with prognosis in multiple cancer types using Oncomine, TIMER, Gene GEPIA2 and CCLE. We examined OSCAR expression correlations with lymph node metastasis and pathological stage across tumor samples using UALCAN and GEPIA2. We analyzed the effects of OSCAR on survival using the Kaplan Meier plotter. We explored genes co-expressed with OSCAR using the LinkedOmics database and analyzed associated gene ontologies using Metascape. Further, we examined the correlation between OSCAR expression and immunocyte infiltration, markers of epithelial-mesenchymal transition, and lymphocyte subtypes using TIMER. OSCAR mRNA levels were upregulated in most cancer types compared with adjacent normal tissues. Higher expression of OSCAR correlated with lymph node metastasis or advanced stage subgroups. High expression of OSCAR was related to low tumor purity, with increased levels of M2 macrophage polarization, T cells exhaustion, and mesenchymal phenotype in most cancer types. We also showed that the strength of OSCAR expression influence in malignant progression and inhibitory immune microenvironment is mitigated by the infiltration of natural killer cells. These findings shed light on the pro-carcinogenic role of OSCAR in most cancer types and indicate OSCAR could be targeted in future therapeutics to reverse the inhibitory immune microenvironment.

## Introduction

Cancer is the second leading cause of death in the United States, and it is becoming the leading cause of death and a major public health problem in China 1, 2. Even though early screening and treatment are the primary anti-tumor strategies (i.e., molecular diagnosis, surgical resection, radiofrequency, chemotherapy, and molecular targeted therapy), the general prognosis still remains extremely dismal in some kinds of cancers, and unfavorable outcomes are attributed to the high frequency of recurrence and metastasis 3. Therefore, continued exploration of new molecules for early prediction and development of new therapeutic strategies are still urgently needed to conquer cancers 4. In recent years, tumor immunotherapy has achieved remarkable efficacy, especially with immune-checkpoint inhibitors showing clinical promise for cancer therapy; however, limited efficacy in certain types of cancer still raise public attention. The urgency remains to explore molecules for the development of new combination therapy strategies.

Previously, our group constructed the oxaliplatin-resistant HCC model, and gene microarray was used to measure changes in gene profile 5. The selection of OSCAR was based on the reanalysis of the different genes in oxaliplatin-resistant HCC tissues. Osteoclast-associated receptor (OSCAR), initially described by Kim et al. as a key molecule in osteoclastogenesis, is considered an interesting potential target to therapeutically inhibit inflammatory osteoclastogenesis 6. It is known that OSCAR is mainly expressed in macrophages, monocytes, and monocyte-derived dendritic cells, and OSCAR has been identified as an important immunological mediator in innate and adaptive immune systems 7. In bone, OSCAR expression serves as costimulatory molecule for osteoclast differentiation, through modulating immune cell interactions 8. Recently, Wu et al. 9 found OSCAR was significantly correlated with overall survival (OS) of lung adenocarcinoma (LUAD), based on the RNAseq data from The Cancer Genome Atlas (TCGA). As most literatures reported, OSCAR was a key molecule in inflammation and immunity orchestration. And inflammation has long been shown to engage in tumor initiation and progression, and numerous immunomodulatory signals are concurrently involved in the regulation of human cancer 10. We speculate that OSCAR might play an important role in the progression of cancer. While, no study had revealed the relationship between OSCAR and human cancers so far.

In the present study, we comprehensively analyzed OSCAR expression and explored its correlation with prognosis in multiple cancer types, using Oncomine, TIMER, GEPIA2, and CCLE. We analyzed the effects of OSCAR on survival using the Kaplan Meier plotter. We examined OSCAR expression correlations with lymph node metastasis and stage across tumor samples using UALCAN and GEPIA2. We explored genes co-expressed with OSCAR using the LinkedOmics database and analyzed associated gene ontologies (GO) using Metascape in multiple cancer types. We also used TIMER to examine the correlation between OSCAR expression and infiltration of immunocytes, lymphocyte subtypes, and makers of epithelial-mesenchymal transition (EMT). Finally, we verified the prediction strengths and related mechanism of OSCAR in glioblastoma (GBM) and skin cutaneous melanoma (SKCM), in which OSCAR played opposing roles in prognosis, using GEPIA2 and TIMER. Our findings suggested that OSCAR facilitates malignancy with enhanced metastasis in multiple types of human cancers correlating to the inhibitory immune microenvironment, and which could be reversed by the infiltration of NK cells.

## Materials and Methods

### OSCAR expression and survival analysis

To analyze the expression level of OSCAR in various cancer types, we used the online database Oncomine, which contains a large collection of independent datasets (https://www.oncomine.org/resource/login.html) 11. The mRNA levels were compared between cancer tissues and their normal tissue counterparts with the following threshold parameters: p<1E-4, fold-change >2, and top gene rank 10%.

OSCAR expression levels in cancer cell lines from diverse cancer types were examined using the Cancer Cell Line Encyclopedia (CCLE) (http://www.broadinstitute.org/ccle), which provides public access to genomic data, analysis, and visualization for more than 1,100 cell lines 12.

GEPIA2 is an updated version of GEPIA for analyzing the RNA sequencing expression data of 9,736 tumors and 8,587 normal samples from the Cancer Genome Atlas (TCGA) and the Genotype Tissue Expression (GTEx) projects, using a standard processing pipeline 13. The RNA-Seq datasets GEPIA2 used is based on the UCSC Xena project (http://xena.ucsc.edu), which are computed by a standard pipeline. OSCAR tumor/normal differential expression and the correlation between expression levels of OSCAR and survival, pathological stage in diverse cancer types were analyzed in GEPIA2.

The Kaplan Meier plotter can be used to assess the effects of 54,000 genes on survival in 21 cancer types. Gene expression data and recurrence free survival (RFS) and overall survival (OS) information were downloaded from Gene Expression Omnibus (GEO), European Genome-phenome Archive (EGA) and TCGA. Correlations between OSCAR expression and survival in diverse types of cancer were analyzed using the Kaplan Meier plotter (http://kmplot.com/analysis/) 14. The hazard ratios (HR) with 95% confidence intervals and log-rank *p*-values were also calculated.

### Clinical features analysis

UALCAN is designed to provide easy access to publicly available cancer OMICS data (TCGA and MET500). It is built on PERL-CGI with high quality graphics using JavaScript and CSS, which is a comprehensive, user-friendly, and interactive web resource for analyzing cancer (http://ualcan.path.uab.edu/index.html) 15. OSCAR expression correlations with lymph node metastasis across tumor samples were analyzed in TCGA analysis module of UALCAN.

### Data collection and screening

GBM, kidney renal clear cell carcinoma (KIRC), low grade glioma (LGG) and Uveal Melanoma (UVM) patients' gene expression profiles, along with their clinical data such as age, gender, grade, tumor stage, TNM classification, and survival status, were downloaded from the TCGA portal (v28.0, https://portal.gdc.cancer.gov/).

### The independent prognostic value of OSCAR

To identify the independent prognostic value of OSCAR in GBM, KIRC, LGG and UVM and assess the correlation between important clinical characteristics and prognosis, we performed Cox analyses. First, we conducted univariate Cox analysis on every variable, in turn, to check their correlation with prognosis. Then, all variables were gathered for a multivariate Cox analysis to evaluate whether each of them has an independent prognostic value. Samples with incomplete data was deleted, and clinical characteristics, in which uncertain or unknown data more than 1/3 in all was excluded. R software was used for Cox analyses.

### Genes co-expressed with OSCAR

Gene co-expression with OSCAR in various cancer types were analyzed using the LinkedOmics database (http://www.linkedomics.org), a publicly available portal that includes multi-omics data from all 32 TCGA Cancer types 16. The top 200 genes with significant positive correlations with OSCAR in different types of cancer were generated by this platform.

### Functional enrichment analysis

Metascape (http://metascape.org) is a free, well-maintained, user-friendly gene-list analysis tool for gene annotation and analysis 17 that functions as an automated meta-analysis tool for understanding common and unique pathways within a group of orthogonal target-discovery studies. In this study, Metascape was used to conduct pathway and process enrichment analysis of the top 200 genes positively correlated with OSCAR in multiple types of cancer. GO terms for biological process, cellular component, and molecular function categories, as well as SKCM pathways, were enriched using the Metascape online tool.

### OSCAR expression and immune cell infiltration in various cancer types

TIMER is a comprehensive resource for systematic analysis of immune infiltrates across diverse cancer types (https://cistrome.shinyapps.io/timer/) 18. Expression levels of OSCAR between tumor and adjacent normal tissues in different types of cancer were identified across all The Cancer Genome Atlas (TCGA) tumors via 'Diff Exp' module in TIMER. The correlation between OSCAR expression and immune infiltration, including B cells, CD4^+^ T cells, CD8^+^ T cells, neutrophils, macrophages, and dendritic cells were explored via 'Gene' modules using TIMER with the associated Spearman's correlation. Spearman correlations were also calculated to evaluate OSCAR expression correlation with various immunocyte, epithelial and mesenchymal makers via 'Correlation' modules in TIMER.

### Statistical analysis

Survival curves were generated by Kaplan Meier plots. Partial heatmaps were drawn using GraphPad Prism version 8 (GraphPad Software, La Jolla, CA, USA). Gene expression correlations were evaluated using Spearman correlations, and p-values <0.05 were considered statistically significant.

## Results

### OSCAR mRNA levels differed in human cancers and OSCAR acted as a risk factor for prognosis in most types of cancers

**Figure 1:**
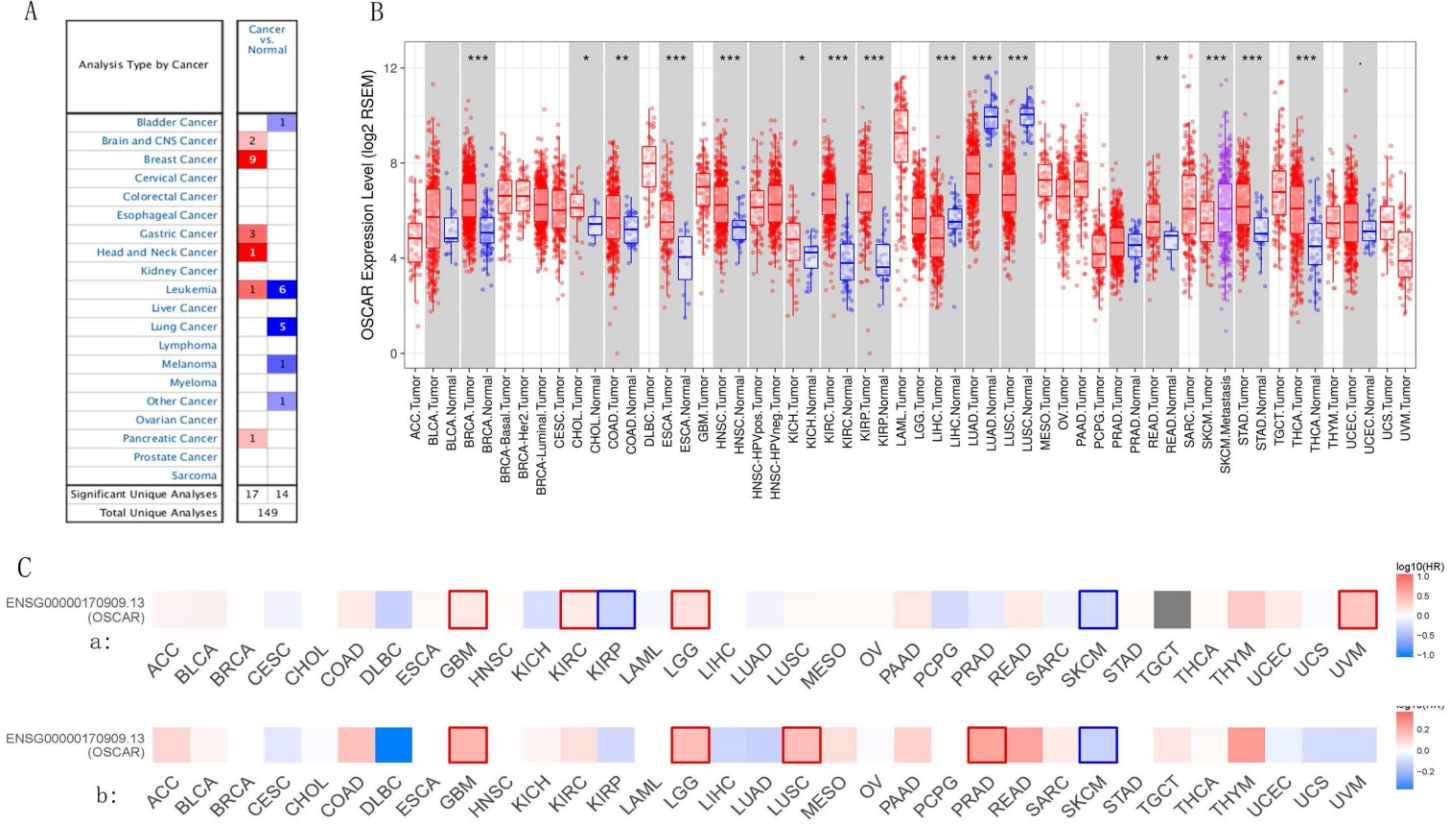
** OSCAR mRNA levels differed in human cancers and OSCAR acted as a risk factor for prognosis in most types of cancers.** (A) OSCAR mRNA levels in different types of human tumors were evaluated using the Oncomine database. (B) OSCAR mRNA levels in 33 types of human tumors were evaluated using TIMER. (C) The prognostic impact of OSCAR, including overall survival (OS, a) and recurrence free survival (RFS, b) were examined using GEPIA 2 in 33 types of cancers.

OSCAR mRNA levels in different types of human tumors were evaluated using the Oncomine database. OSCAR mRNA levels in cancer compared to healthy tissues revealed that OSCAR expression was higher in brain and central nervous system cancers, breast cancer (BRCA), gastric cancer, head and neck cancer (HNSC), leukemia, and pancreatic adenocarcinoma (PAAD), compared with adjacent normal tissues. However, OSCAR expression was significantly lower in bladder cancer (BLCA), leukemia (different subtypes), lung cancer, and melanoma, compared with adjacent normal tissues (Fig. 1A). Additionally, using the TIMER database to further evaluate the RNA-Seq data, expression of OSCAR was found to be significantly upregulated in 11 types of human cancers, including BRCA, cholangiocarcinoma (CHOL), colon adenocarcinoma (COAD), esophageal cancer (ESCA), head and neck cancer (HNSC), kidney chromophobe, KIRC, kidney renal papillary cell carcinoma (KIRP), rectal adenocarcinoma (READ), stomach adenocarcinoma (STAD), and thyroid carcinoma (THCA), compared with the associated normal tissues. Only in liver hepatocellular carcinoma (LIHC), lung adenocarcinoma (LUAD), and lung squamous cell carcinoma (LUSC), was OSCAR expression significantly lower than that in adjacent normal tissues (Fig. 1B). In addition, OSCAR expression levels in cancer cell lines was assessed in CCLE datasets (Supp. Fig. 1A). These results revealed that the mRNA levels of OSCAR were differed in human cancers and the levels of which were significantly upregulated in multiple cancer types compared with normal tissues.

We investigated whether OSCAR expression was correlated with prognosis in cancer patients. The relationship of OSCAR expression and survival rate was evaluated using the GEPIA2 database in 33 human cancers. In most types of cancers, OSCAR expression correlated with poorer prognosis, including as BLCA, BRCA, COAD, ESCA, GBM, HNSC, KIRC, LUSC, LGG, READ, and STAD (Fig. 1C). Only in a small number of cancer types, such as cervical and endocervical carcinoma (CESC), KIRP, LUAD, SKCM, and uterine corpus endometrial carcinoma (UCEC), did OSCAR expression implicate a protective role, as high expression was associated with better prognosis for these cancer types (Fig.1C). For further confirmation of this finding, Kaplan Meier plots were used to analyze survival in human cancers. The patients in the OSCAR-high group had shorter OS than those in the OSCAR-low group for BLCA, KIRC, THYM, and patients in the OSCAR-high group also had shorter RFS than those in the OSCAR-low group for ESCA, HNSC, and PAAD with significant statistical differences (Supp. Fig. 1B). Together these findings suggest that OSCAR acted as a risk factor for prognosis in most types of cancers, and the predictive strength varied for diverse types of cancer.

### OSCAR mRNA levels were higher in cancer subgroups with higher malignancy

**Figure 2:**
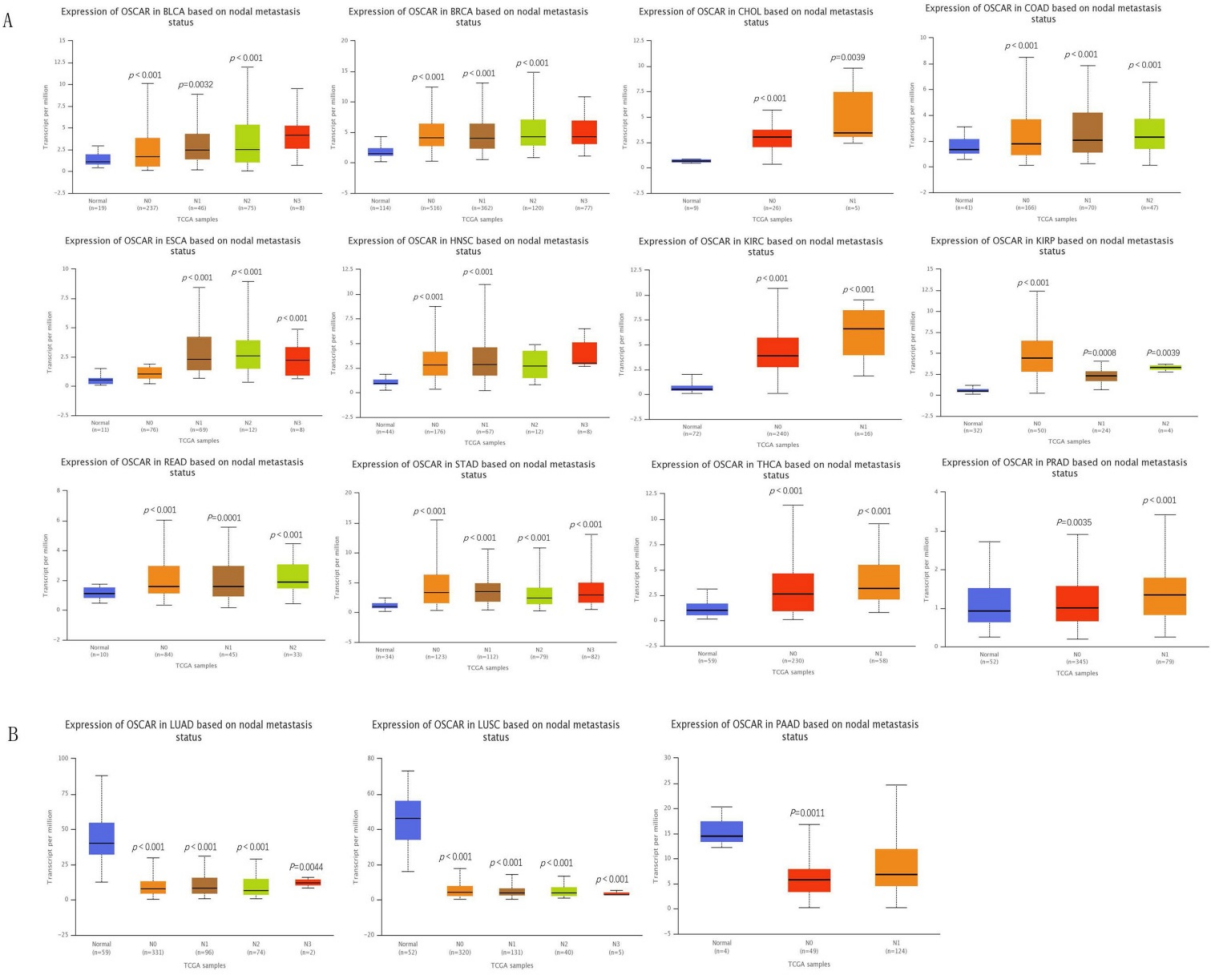
** OSCAR protein expression was higher in subgroup of cancers with metastasis.** The expression of OSCAR in different types of cancer based on nodal metastasis status using a box plot in UALCAN database. (A) OSCAR-high with higher nodal status (12 cancers). (B) OSCAR-low with higher nodal status (3 cancers).

We profiled the expression of OSCAR in different types of cancer based on metastasis status using a box plot in UALCAN database. First, we enrolled 25 cancers, metastasis data were missing for 4 of these cancers, and 6 of these cancers were with no difference. Therefore, analysis was conducted on the remaining 15 cancer species. Twelve types of cancer patients in the OSCAR-high group (BLCA, BRCA, CHOL, COAD, ESCA, HNSC, KIRC, KIRP, PRAD, READ, STAD, THCA) had higher metastasis status than those in the OSCAR-low group, with statistically significant differences (Fig. 2A). Three types of cancer patients had metastasis status in the OSCAR-low group, including LUAD, LUSC, and PAAD (Fig. 2B).

Upon further study, we found significant differences in OSCAR expression in different stages using GEPIA 2. There is a positive correlation between the expression of OSCAR and cancer stages. (Supp. Fig. 2A). These findings revealed that OSCAR protein expression was higher in subgroup of cancers with higher malignancy.

### High expression of OSCAR is a potential independent risk factor

To find more evidence, the Cox proportional-hazards model was constructed in GBM, KIRC, LGG and UVM, which have significant results in OS significance map of OSCAR from GEPIA2. The clinical characteristics of GBM, KIRC, LGG and UVM were obtained from the TCGA datasets, including age, gender, grade, tumor stage and TNM classification (Supp. Table 1). Univariate Cox analyses in OS showed that age and OSCAR in GBM; age, grade, stage, T classification, M classification and OSCAR in KIRC; age, grade and OSCAR in LGG; and stage in UVM were acting potential risk roles. Additionally, the multivariate Cox analyses confirmed the critical value of age and OSCAR in GBM; age, grade and stage in KIRC; age, grade and OSCAR in LGG; and stage in UVM, proving that they can predict tumor prognosis independently of other factors in OS (Table 1). Taking together, OSCAR is a potential marker to predict survival.

### Genes co-expressed with OSCAR were mainly associated with leukocyte activation and immunocyte infiltration

**Figure 3:**
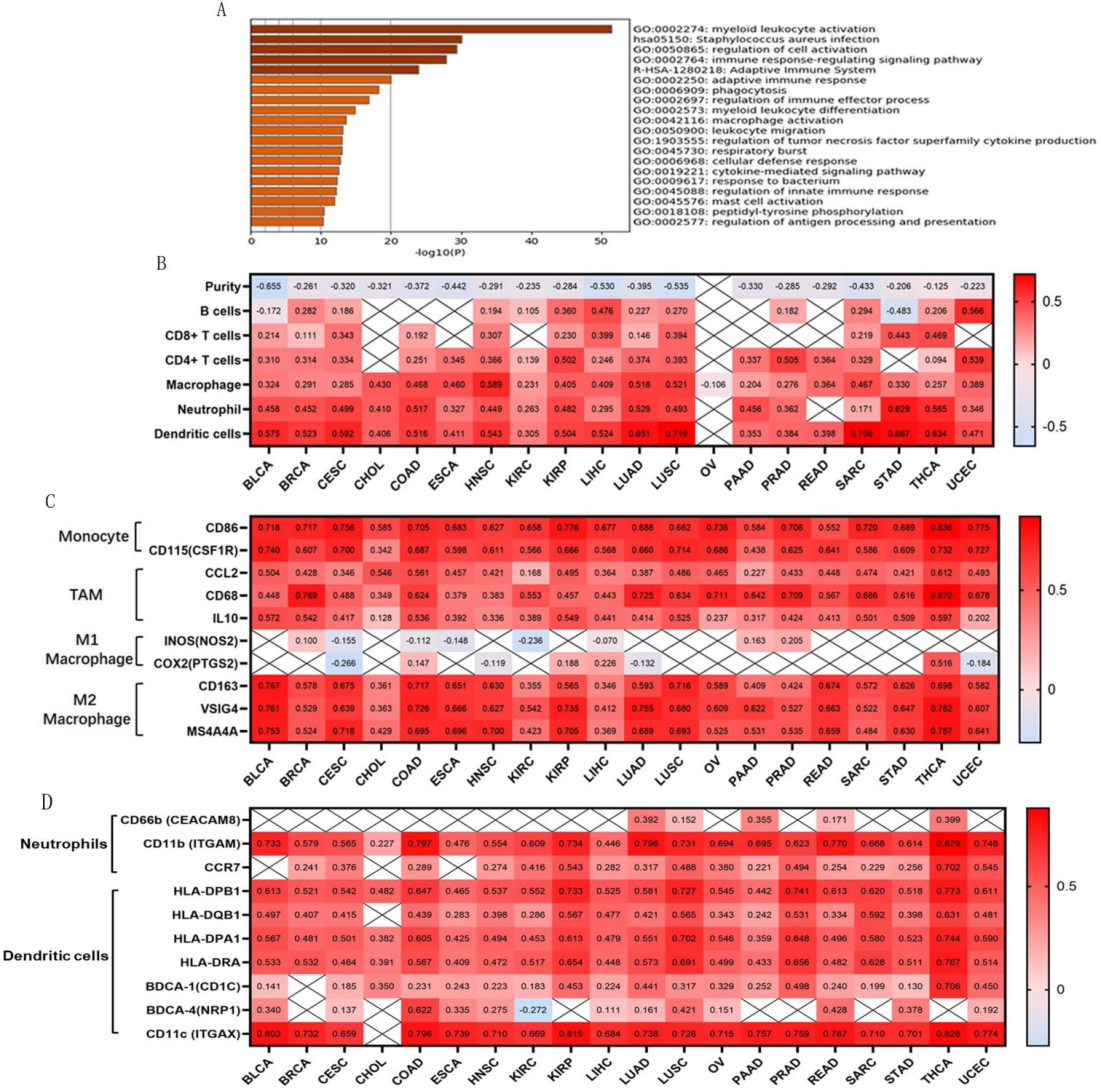
** Genes co-expressed with OSCAR were mainly associated with leukocyte activation and immunocyte infiltration.** (A) Genes co-expressed with OSCAR in the selected in various cancer types were explored using the LinkedOmics database. The top 200 related genes were included, and the top 20 clusters with enriched terms (GO/KEGG terms) were visualized using Metascape. (B) Correlation coefficients for the 20 cancers studied; OSCAR expression was closely correlated with tumor purity and immunocyte infiltration levels as evaluated by TIMER. (C) The correlation coefficients between OSCAR and markers of monocytes, TAMs, and M1 and M2 macrophages in diverse cancer types using TIMER. (D) OSCAR expression was also showed strong correlations with infiltrating levels of neutrophils and dendritic cells, verified by a variety of surface markers using TIMER, in the selected 20 types of cancers.

The top 200 related genes co-expressed with OSCAR were selected from all 32 TCGA Cancer types and explored using the LinkedOmics database. Accumulative hypergeometric *p*-values and enrichment terms were calculated, exhibiting in the form of heatmap, and the top 20 clusters with enriched terms (GO/KEGG terms) were visualized using Metascape. We found that the genes co-expressed with OSCAR were significantly enriched in GO for myeloid leukocyte activation in these 32 types of cancers (Fig. 3A). Therefore, we speculated that OSCAR affected malignant progression mainly through the dialogue with the immune.

Tumor-infiltrating lymphocytes are an independent predictor of cancer prognosis 19. Therefore, we included 20 cancers for which OSCAR expression was closely related to prognosis to investigate correlations between OSCAR expression, tumor purity, and immunocyte infiltration levels using TIMER. We found negative correlations between OSCAR expression and tumor purity as well as varying directions and strengths of correlations between OSCAR and immunocyte infiltration. OSCAR expression was significantly correlated with infiltrating levels of B cells in 14/20 types of cancer, CD8^+^ T cells in 12/20 types of cancer, CD4^+^ T cells in 17/20 types of cancer, macrophages in 20/20 types of cancer, neutrophils in 18/20 types of cancer, and dendritic cells in 19/20 types of cancer (Fig. 3B). These results reveal strong correlations between OSCAR and immunocyte infiltration, with particularly strong positive correlations with macrophages.

To further investigate correlations between OSCAR and macrophage subtypes in multiple cancer types, we focused on markers of monocytes, tumor-associated macrophages (TAMs), and M1 and M2 macrophages using TIMER. OSCAR expression was positively correlated with infiltrating levels of monocytes in 20/20 types of cancer, especially BLCA (CD86, r=0.718; CD115, r=0.740), CESC (CD86, r=0.756; CD115, r=0.700), THCA (CD86, r=0.836; CD115, r=0.732), and UCEC (CD86, r=0.775; CD115, r=0.727). In addition, OSCAR expression was positively correlated with infiltrating levels of TAMs in 20/20 types of cancer, especially BRCA (CCL2, r=0.428; CD68, r=0.769; IL10, r=0.542), COAD (CCL2, r=0.561; CD68, r=0.624; IL10, r=0.536), and THCA (CCL2, r=0.612; CD68, r=0.870; IL10, r=0.597). Finally, OSCAR expression was positively correlated with infiltrating levels of M2 macrophages in 20/20 types of cancer, especially BLCA (CD163, r=0.767, VSIG4, r=0.761, MS4A4A r=0.753), COAD (CD163, r=0.717, VSIG4, r=0.726, MS4A4A, r=0.695), and THCA (CD163, r=0.698, VSIG4, r=0.782, MS4A4A r=0.787). Correlations between OSCAR expression and M1 macrophages were weak or non-existent (Fig. 3C). OSCAR expression also showed a strong correlation with infiltrating levels of neutrophils and dendritic cells, verified by a variety of surface markers, in the selected 20 types of cancers (Fig. 3D).

### OSCAR was positively associated with T cells exhaustion and mesenchymal phenotype in diverse human cancers

**Figure 4:**
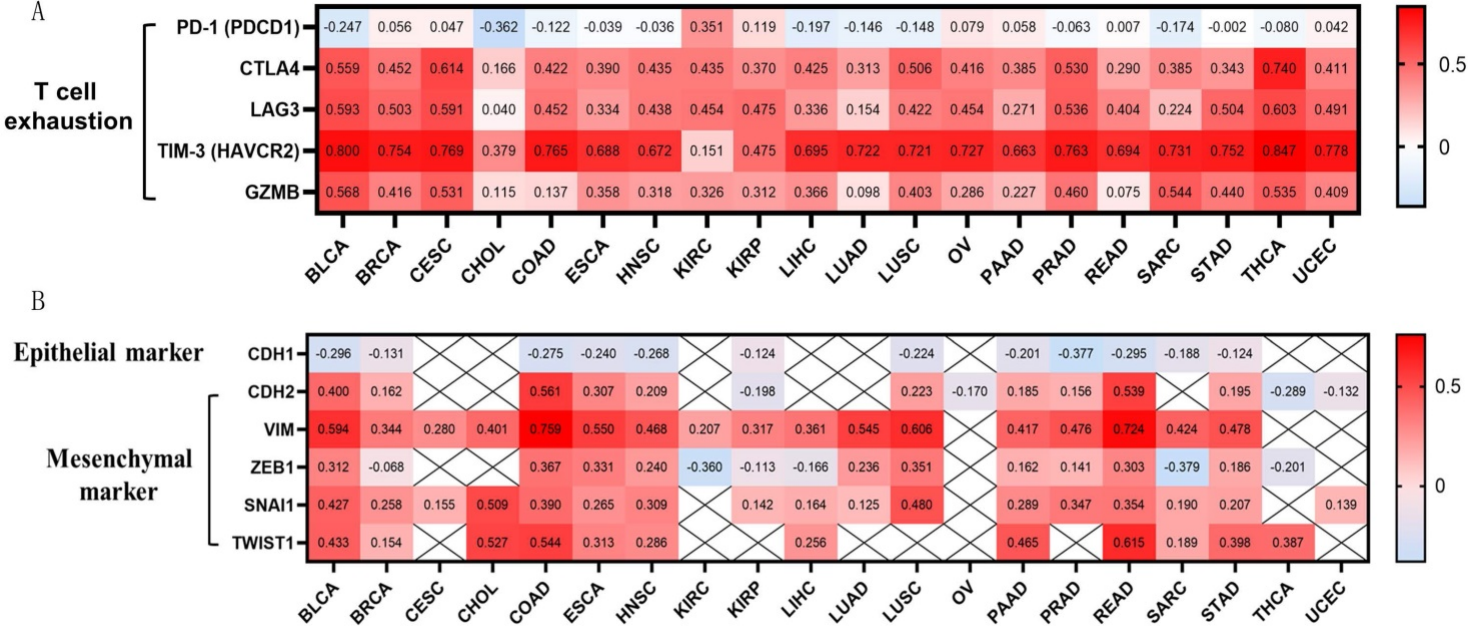
** OSCAR was positively associated with T cells exhaustion and mesenchymal phenotype in diverse human cancers.** (A) OSCAR expression showed significant positive correlation with CTLA-4, LAG3, TIM-3, and GZMB, revealed high expression of OSCAR promoting T cells exhaustion. (B)The positive correlations between OSCAR and markers of EMT were investigated in 20 types of cancer using TIMER.

OSCAR expression showed a significant positive correlation with CTLA-4, LAG3, TIM-3, and granzyme B (GZMB), and we also found that high expression of OSCAR promoted T cells exhaustion (Fig. 4A). The correlations between OSCAR and markers of natural killer (NK) cell and T cell subtypes in multiple cancer types was also generated by TIMER (Supp. Fig.3). These findings indicate that high expression of OSCAR was correlated to low tumor purity, with increased levels of M2 macrophage polarization, and T cell exhaustion.

EMT is a critical process in the pathogenesis of metastasis 20. To further investigate correlations between OSCAR and EMT in diverse cancer types, we focused on the marker of epithelial phenotype CDH1 (E-cadherin), and markers of mesenchymal phenotype, CDH2 (N-cadherin), Vim (vimentin), and transcription factors (ZEB1, TWIST1, and Snail) in 20 types of cancer using TIMER. The expression levels of CDH1 were negatively correlated with OSCAR in 12/20 types of cancer, especially BLCA (r=-0.296), COAD (r=-0.275), PRAD (r=-0.377), and READ (r=-0.295) (Fig. 4B). Meanwhile, we found that OSCAR expression was positively correlated with multiple markers of mesenchymal phenotype in diverse types of cancer. Vimentin is a typical marker of mesenchymal phenotype, which was positively correlated with the levels of OSCAR in 17/20 types of cancer, especially BLCA (r=0.594), COAD (r=0.759), LUSC (r=0.606), and READ (r=0.724). We also found positive correlations between OSCAR expression and other markers of mesenchymal phenotype in varying strengths (Fig. 4B). These findings indicate that high expression levels of OSCAR were positively associated with mesenchymal phenotype in diverse human cancers.

### The strength of OSCAR activity in inhibitory immune microenvironment was mainly reversed by the infiltration of NK cells

**Figure 5:**
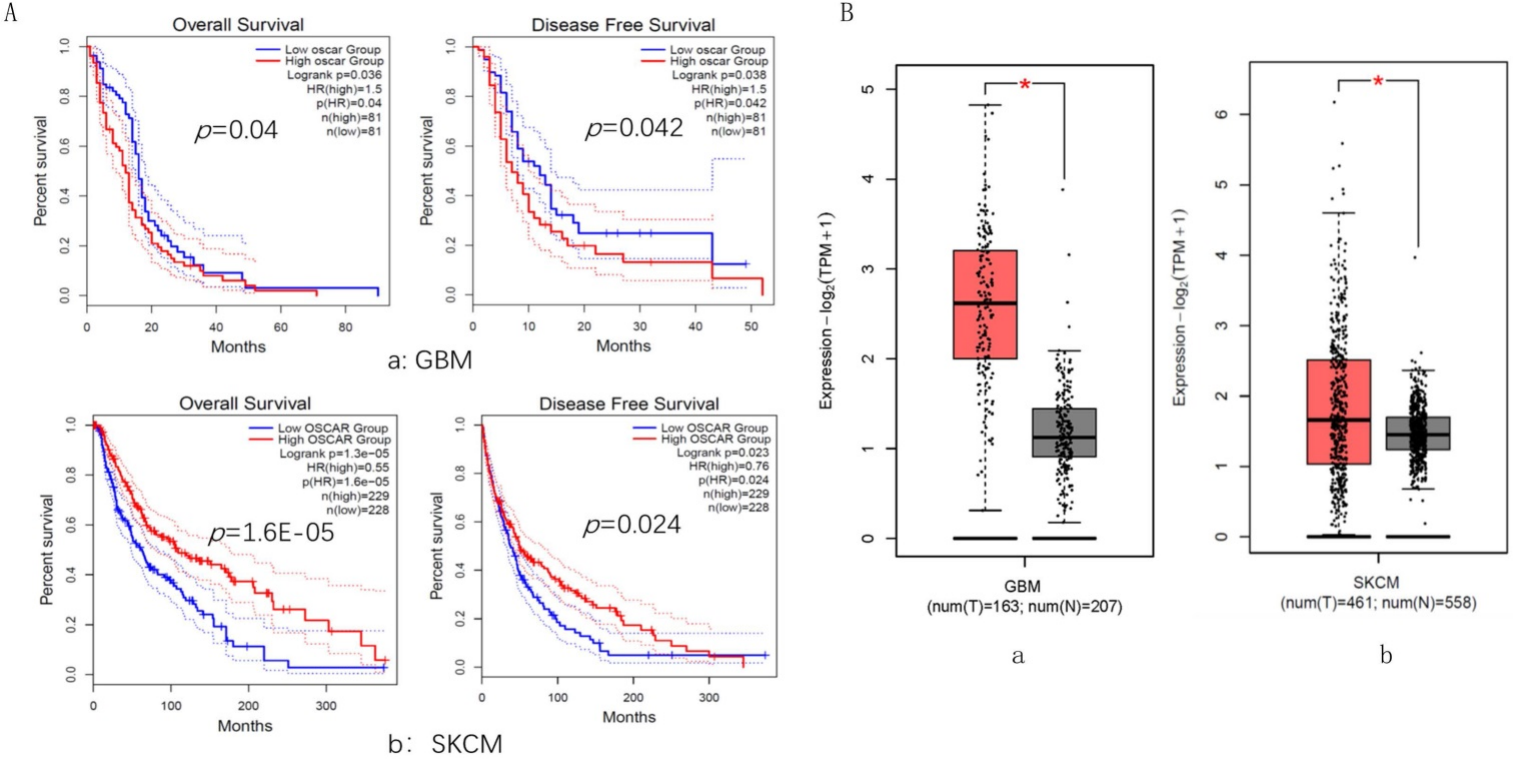
** The expression and role in prognosis of OSCAR were explored in glioblastoma (GBM) and skin cutaneous melanoma (SKCM).** OSCAR expression levels played an opposite role in prognosis in GBM and SKCM, evaluated by GEPIA 2. (B) OSCAR mRNA levels in cancer were higher in both GBM and SKCM, compared to healthy tissues.

GBM and SKCM cancer types were selected, in which their OSCAR expression levels played an opposite role in prognosis. For GBM, the OSCAR-high group had shorter OS (HR=1.5, *p*=0.040) and RFS (HR=1.5, *p*=0.042) than those in the OSCAR-low group (Fig. 5A, a). In contrast for SKCM, OSCAR had a protective role, as high expression was associated with longer OS (HR=0.55, *p*=0.000) and RFS (HR=0.76, *p*=0.024, Fig. 5A, b). OSCAR mRNA levels in cancer compared to healthy tissues revealed that OSCAR expression was higher in both GBM (p<0.05, Fig. 5B, a) and SKCM (p<0.05, Fig. 5B, b).

**Figure 6:**
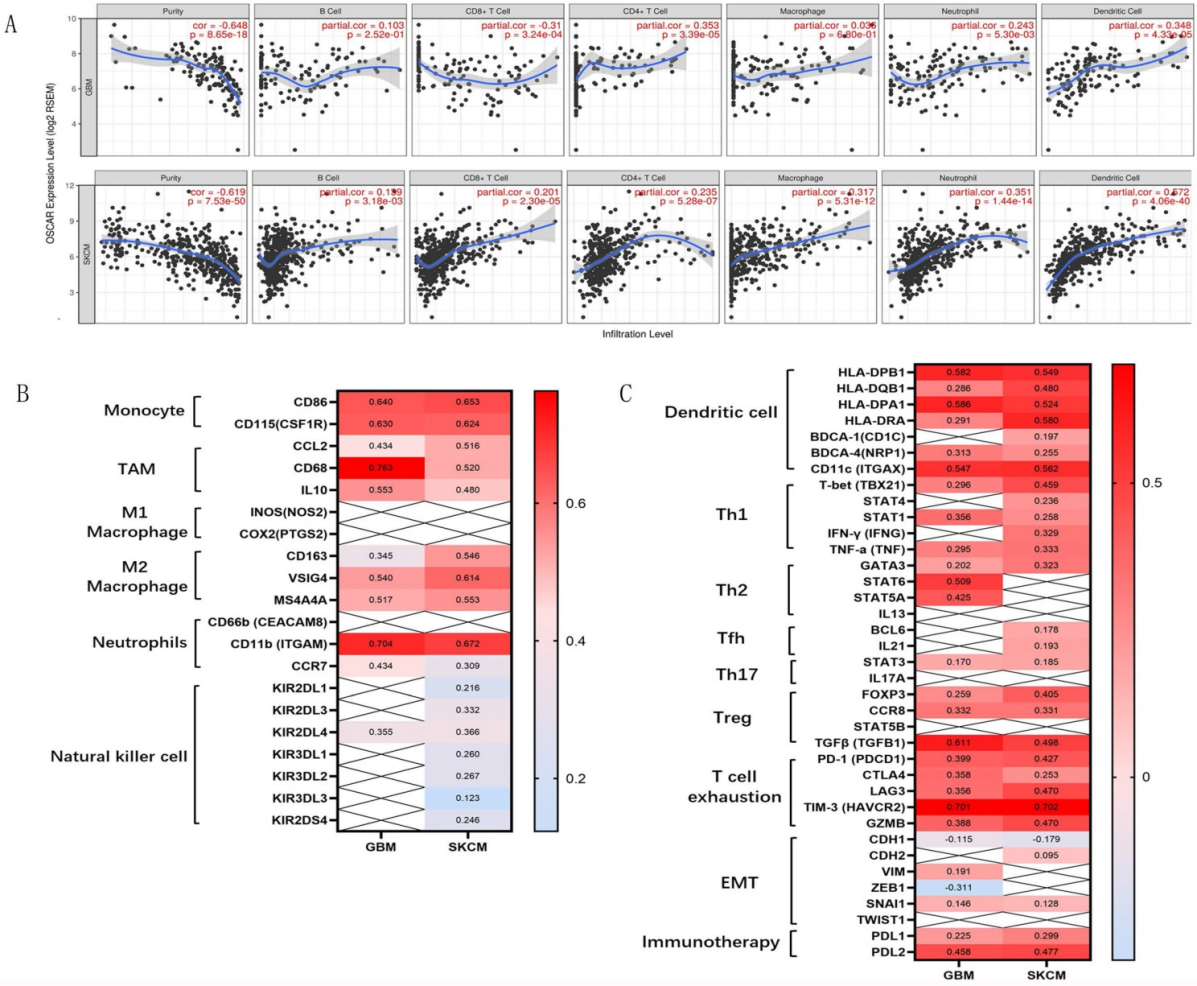
** The strength of OSCAR expression in malignant progression and immunocyte infiltration were diverse in GBM and SKCM.** (A) The correlations between OSCAR expression and tumor purity and immunocyte infiltration in GBM and SKCM as evaluated by TIMER. (B) The correlations between OSCAR expression and immune cell subsets, including B cells, various macrophage subtypes, NK cells were evaluated by TIMER and expressed by correlation coefficients. (C) The correlations between OSCAR expression and dendritic cell subsets, T cell subtypes, and markers of EMT were investigated using TIMER and expressed by correlation coefficients.

Further analyzing the mechanism behind this paradox, we found strongly negative correlations between OSCAR expression and tumor purity in GBM (r=-0.648) and SKCM (r=-0.619; Fig. 6A). Meanwhile, OSCAR expression was weakly correlated with infiltrating levels of B cells, CD8^+^ T cells, CD4^+^ T cells, neutrophils, and dendritic cells in GBM and SKCM. As to macrophages, there was no significant correlation with OSCAR in GBM (r=0.036, *p*=0.68), and moderate correlation with OSCAR in SKCM (r=0.317, p=0.00; Fig. 6A). To further investigate the role of immune cell subsets in malignant progression, we found that there was no difference in M2 macrophage, Th1 cells and T cells exhaustion between GBM and SKCM. And there was also no difference in the infiltrating levels of dendritic cells and Treg cells between GBM and SKCM (Fig. 6B and Fig. 6C). NK cell-mediated innate immunity is necessary for the tumor growth inhibition effects 21. OSCAR expression in SKCM showed significant correlation with infiltrating levels of NK cells, as verified by surface markers including KIR2DL1 (r=0.216), KIR2DL3 (r=0.332), KIR2DL4 (r=0.216), KIR3DL1 (r=0.260), KIR3DL2 (r=0.267), KIR3DL3 (r=0.123), and KIR2DS4 (r=0.246; Fig. 6B). In contrast for GBM, there was only one correlation between OSCAR and KIR2DL4 (r=0.355; Fig. 6B). Together these findings suggest that the strength of OSCAR activity in malignant progression and the inhibitory immune microenvironment was influenced by the infiltration of NK cells in certain types of cancer.

## Discussion and Conclusion

In human cancer, a series of genetic and phenotypic changes and the corresponding cytokines, chemokines and metabolites derived from tumor cells have a significant impact on the infiltration of diverse types of stromal cells; together these factors comprise the tumor microenvironment (TME) 22. TME mainly marked by lymphocytes, vascular endothelial cells, and fibroblast involvement, and which orchestrate the process of malignant transformation into a permissive state that promotes tumor progression 23. Solid tumors that appear in sites such as head and neck, lung, breast, liver, stomach, colon, prostate, and skin are constructed by different proportions and types inflammatory cells, endowing tumor progenitors with diverse malignant features and drug responses 24. Thus, the identification of effective factors that regulate the immune microenvironment is an important and effective approach for predicting the survival and improving treatment efficacy in cancer patients.

OSCAR, an IgG-like receptor, was described as a key molecule contributing to the pathogenesis and severity of osteoporosis and rheumatoid arthritis 7. OSCAR is part of the costimulatory domain of numerous immunomodulatory molecules and signals through integrin subunit alpha M (ITAM) motifs and the common γ-chain of the Fc receptor. OSCAR expression is commonly involved in the regulation of bone resorption and bone formation, and the equilibrium requires fine-tuning mechanisms 25. During the tumor progression, tumor-infiltrating immune cells such as regulatory T cells, TAMs, and myeloid-derived suppressor cells secrete inflammatory cytokines to promote angiogenesis and tumor cell proliferation, invasion and metastasis in a negative-tuning mechanisms 26. Therefore, we speculate that OSCAR plays an important role in the progression of cancer. In the present study, expression levels of OSCAR between tumor and adjacent normal tissues in different types of cancer were identified across all TCGA tumors in TIMER, and we showed OSCAR was significantly upregulated in most types of human cancers. GEPIA 2 and UALCAN databases revealed higher expression of OSCAR mainly in cancer patients with advanced stages and higher nodal metastasis statuses. Cox analyses revealed that high expression of OSCAR is a potential a potential marker to predict survival. Further research using TIMER revealed that high expression of OSCAR was positively associated with mesenchymal phenotype in diverse human cancers, with upregulated mesenchymal markers (CDH2, Vimentin, Snail1, Twist1, etc.) and downregulated epithelial maker of E-cadherin. Together these findings suggested that OSCAR protein expression was higher in subgroups of cancers with higher malignancy.

Activation of OSCAR signaling increases the production of chemokines such as monocyte chemoattractant protein 1 (MCP-1), which is involved in the recruitment of leukocytes sites of inflammation 25. Barrow et al. reported that OSCAR was expressed on the cell surface of lung interstitial myeloid cells and blood monocytes, which stimulates the secretion of TNF-α 27. To further analyze the role of OSCAR in regulating the tumor microenvironment, the top 200 genes co-expressed with OSCAR were examined in 32 TCGA Cancer types using the LinkedOmics database, and enriched GO/KEGG terms were explored using Metascape. The results revealed that genes co-expressed with OSCAR were mainly enriched in GO of myeloid leukocyte activation. Using TIMER, we revealed strong correlations between OSCAR and immunocyte infiltration, with particularly strong positive correlations with macrophages, dendritic cells, and T cells. To further investigate the correlations between OSCAR in leukocyte subtypes in cancer types, we showed that high expression of OSCAR was related to low tumor purity, with increased levels of M2 macrophage polarization and T cell exhaustion. So we showed that OSCAR facilitates malignancy with enhanced metastasis involving the inhibitory immune microenvironment, including M2 macrophage polarization and T cell exhaustion.

The diverse state of immunocyte infiltration in different types of cancer attributed to the diverse predictive strength, or even to an opposite role in prognosis 28. We investigated whether OSCAR expression was correlated with prognosis and demonstrated that OSCAR expression was highly correlated with poorer outcomes in most types of cancers; however, the predictive strength of OSCAR expression varied for different types of cancer. And in other cancer types, their OSCAR expression levels even played an opposite role in prognosis. To further clarify this phenomenon, 2 types of cancer GBM and SKCM were selected. In GBM and SKCM, OSCAR mRNA levels were both higher in cancer compared to healthy tissues, while the correlations of OSCAR expression to prognosis were contradictory. Upon further analysis of the mechanism behind this paradox, we found for both GBM and SKCM the same correlations between OSCAR expression and tumor purity, infiltrating levels of B cells, CD8^+^ T cells, CD4^+^ T cells, neutrophils, and dendritic cells, and the macrophages subtypes. Interestingly, OSCAR expression in SKCM demonstrated a significant correlation with infiltrating levels of NK cells, while the correlation between OSCAR and NK cell infiltration for GBM was weak or negative. Tumor-infiltrating lymphocytes are an independent predictor of cancer prognosis, and NK-mediated innate immunity is necessary for the tumor growth inhibition effects 29. NK cells are a heterogeneous subset of immune cells, which express a diverse array of activating and inhibitory germline-encoded receptors, and are thus capable of directly targeting and killing cancer cells without the need for MHC specificity 30. And we believed that the strength of OSCAR expression correlation with malignant progression and inhibitory immune microenvironment was partly mitigated by the infiltration levels of NK cells.

We propose that high expression of OSCAR is largely associated with poorer prognosis for many cancers, serving as a biomarker when considered alongside inhibitory immune microenvironment. Further the strength of OSCAR expression influence on malignant progression and the inhibitory immune microenvironment is partly mitigated by the infiltration of NK cells in certain types of cancer. These findings shed light on the pro-carcinogenic role of OSCAR in multiple cancer types and indicate that OSCAR could potentially be targeted in future therapies aimed at reversing the inhibitory immune microenvironment. While our study was only limited to bioinformatics analysis, and lack of lack of *in vitro* and *in vivo* deeper mechanistic verification. Therefore, several fundamental questions remain to be answered concerning the role and mechanism in the further study.

## Supplementary Material

Supplementary figures and tables.

## Figures and Tables

**Table 1:** Univariate analysis and multivariate analysis of the correlation of OSCAR expression and important clinical characteristics with OS among patients in the GBM, KIRC, LGG and UVM

Parameter	Univariate analysis			Multivariate analysis		
	HR	95% CI	*P*-value	HR	95% CI	*P*-value
**GBM (N=159)**						
Age	1.0330	1.0161-1.050	**1.141E-04**	1.0347	1.017-1.052	**7.213E-05**
Gender	0.869	0.590-1.279	4.770E-01	0.931	0.629-1.380	7.23E-01
OSCAR	1.089	1.023-1.159	**7.522E-03**	1.102	1.036-1.173	**2.114E-03**
**KIRC (N=489)**						
Age	1.033	1.019-1.047	**2.292E-06**	1.036	1.021-1.051	**3.359-E06**
Gender	0.931	0.675-1.284	6.629E-01	0.998	0.718-1.388	9.914E-01
Grade	2.293	1.854-2.836	**1.939E-14**	1.488	1.165-1.901	**1.443E-03**
Stage	1.889	1.649-2.164	**4.671E-20**	1.696	1.086-2.649	**2.012E-02**
T classification	1.941	1.639-2.299	**1.501E-14**	0.861	0.572-1.296	4.734E-01
M classification	4.284	3.106-5.908	**7.450E-19**	1.265	0.646-2.479	4.925E-01
OSCAR	1.158	1.084-1.237	**1.321E-05**	1.059	0.977-1.148	1.606E-01
**LGG (N=508)**						
Age	1.065	1.048-1.081	**1.561E-15**	1.061	1.044-1.078	**3.625E-13**
Gender	1.060	0.726-1.548	7.619E-01	1.099	0.746-1.619	6.339E-01
Grade	3.120	2.061-4.724	**7.445E-08**	2.432	1.587-3.726	**4.469E-05**
OSCAR	1.183	1.099-1.274	**8.497E-06**	1.133	1.058-1.213	**3.523E-04**
**UVM (N=79)**						
Gender	2.024	0.631-6.484	2.355E-01	1.722	0.529-5.604	3.664E-01
Stage	6.160	1.731-21.919	**4.992E-03**	9.816	2.126-45.327	**3.433E-03**
T classification	1.875	0.765-4.598	1.695E-01	0.528	0.165-1.691	2.819E-01
OSCAR	1.352	0.671-2.725	3.992E-01	1.170	0.559-2.449	6.770E-01

GBM, Glioblastoma multiforme; KIRC: Kidney renal clear cell carcinoma; LGG: Brain Lower Grade Glioma; UVM: Uveal Melanoma; HR: hazard ratio; CI: confidence interval. Bold values indicate *p*-value < 0.05.

## Abbreviations

BLAD: bladder cancer
BRCA: breast cancer
CCLE: Cancer Cell Line Encyclopedia
CESC: cervical and endocervical carcinoma
CHOL: cholangiocarcinoma
COAD: colon adenocarcinoma
EGA: European Genome-phenome Archive
EMT: epithelial-mesenchymal transition
ESCA: esophageal cancer
GBM: glioblastoma
GEO: Gene Expression Omnibus
GEPIA2: Gene Expression Profiling Interactive Analysis 2
GO: gene ontologies
GTEx: Genotype Tissue Expression
GZMB: granzyme B
HNSC: head and neck cancer
HR: hazard ratios
ITAM: integrin subunit alpha M
KEGG: Kyoto Encyclopedia of Genes and Genome
KIRC: kidney renal clear cell carcinoma
KIRP: kidney renal papillary cell carcinoma
LGG: low grade glioma
LIHC: liver hepatocellular carcinoma
LUAD: lung adenocarcinoma
LUSC: lung squamous cell carcinoma
MCP-1: monocyte chemoattractant protein 1
NK: Natural killer
S: overall survival
OSCAR: osteoclast-associated
PAAD: pancreatic adenocarcinoma
READ: rectal adenocarcinoma
RFS: recurrence free survival
SKCM: skin cutaneous melanoma
STAD: stomach adenocarcinoma
TAMs: tumor-associated macrophages
TCGA: the Cancer Genome Atlas
THCA: thyroid carcinoma
TIMER: Tumor IMmune Estimation Resource
TME: tumor microenvironment
UCEC: uterine corpus endometrial carcinoma
