# Supplementary material for: OSCAR facilitates malignancy with enhanced metastasis correlating to inhibitory immune microenvironment in multiple cancer types
Source: J Cancer. 2021 May 5;12(13):3769–80. doi: 10.7150/jca.51964 (PMC8176254; doi:10.7150/jca.51964)
Supplement: Supplementary file 1 — Supplementary figures and tables. [file jcav12p3769s1.pdf]

Supp. table 1. The characteristics of patients in the GBM, KIRC, LGG and UVM

	GBM		KIRC		LGG		UVM	
Characteristic	Total (N=599)	Percentage (%)	Total (N=537)	Percentage (%)	Total (N=515)	Percentage (%)	Total (N=80)	Percentage (%)
**Age**								
≤60	326	54.42	266	49.53	454	88.16		
> 60	273	45.58	271	50.47	61	11.84		
unknown							80	100
**Gender**								
Female	230	38.40	191	35.57	230	44.66	35	43.75
Male	369	61.60	346	64.43	285	55.34	45	56.25
**Grade**								
G1			14	2.61				
G2			230	42.83	249	48.35		
G3			207	38.55	265	51.46		
G4			78	14.53				
Gx			5	0.93				
unknown	599	100	3	0.56	1	0.19	80	100
**Tumor stage**								
Stage I			269	50.09				
Stage II			57	10.61			39	48.75
Stage III			125	23.28			36	45.00
Stage IV			83	15.46			4	5.00
unknown	599	100	3	0.56	515	100	1	1.25
**T classification**								
T1			275	51.21				
T2			69	12.85			14	17.50
T3			182	33.89			32	40.00
T4			11	20.48			34	42.50
unknown	599	100			515	100		
**N classification**								
N0			240	44.69			52	65.00
N1			17	3.17				
Nx			280	52.14			27	33.75
unknown	599	100					1	1.25
**M classification**								
M0			426	79.33			51	63.75
M1			79	14.71			4	5.00
Mx			30	5.59			23	28.75
unknown	599	100	2	0.37	515	100	2	2.5

GBM, Glioblastoma multiforme; KIRC: Kidney renal clear cell carcinoma; LGG: Brain Lower Grade Glioma; UVM: Uveal Melanoma.

Supp. fig. 1

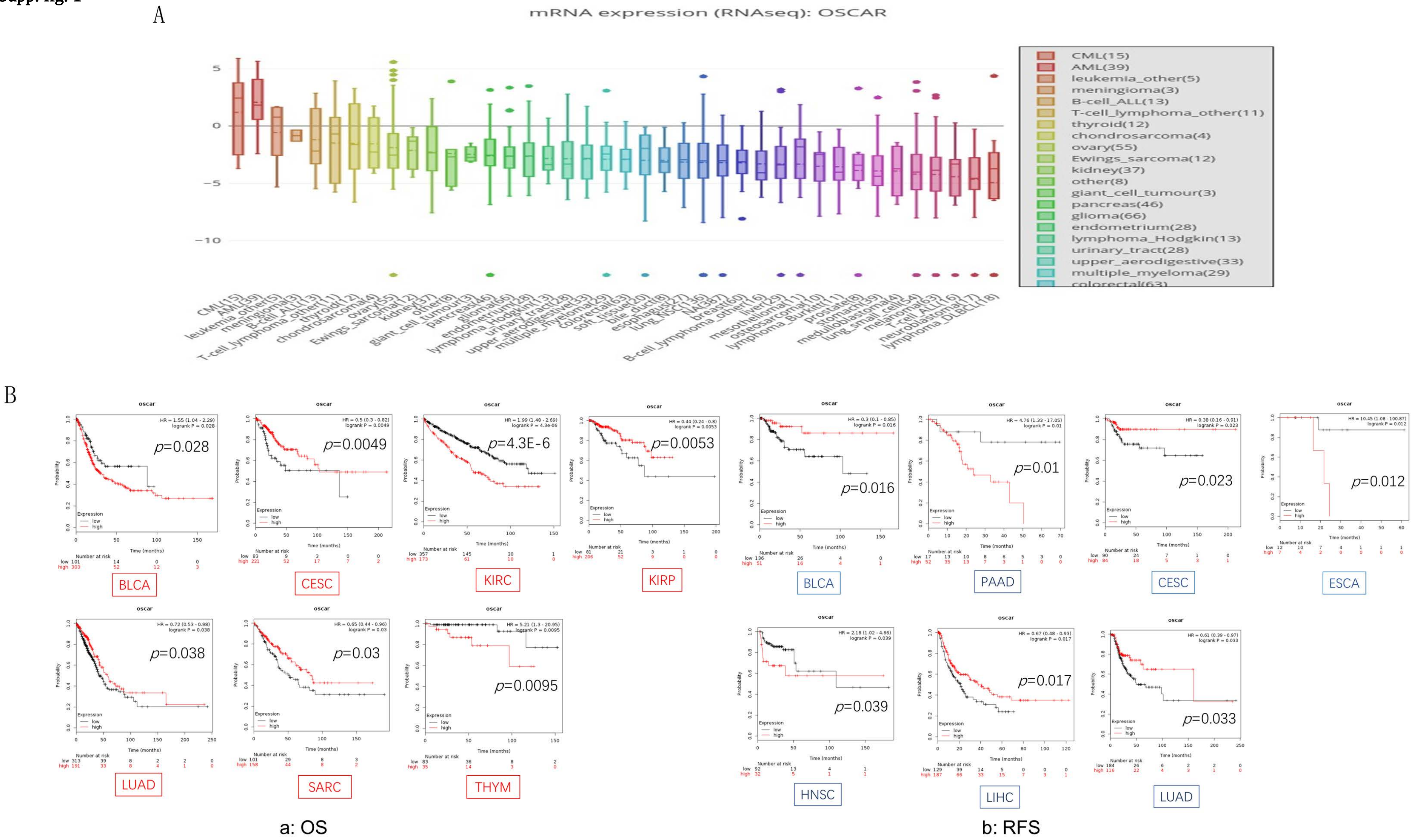
A
mRNA expression (RNAseq): OSCAR
CML(15)
AML(39)
leukemia_other(5)
meningioma(3)
B-cell_ALL(13)
T-cell_lymphoma_other(11)
thyroid(12)
chondrosarcoma(4)
ovary(55)
Ewings_sarcoma(12)
kidney(37)
other(8)
giant_cell_tumour(3)
pancreas(46)
glioma(66)
endometrium(28)
lymphoma_Hodgkin(13)
urinary_tract(28)
upper_aerodigestive(33)
multiple_myeloma(29)
B
p=0.028
BLCA
p=0.0049
CESC
p=4.3E-6
KIRC
p=0.0053
KIRP
p=0.016
BLCA
p=0.01
PAAD
p=0.023
CESC
p=0.012
ESCA
p=0.038
LUAD
p=0.03
SARC
p=0.0095
THYM
p=0.039
HNSC
p=0.017
LIHC
p=0.033
LUAD
a: OS
b: RFS

Supp. fig. 2

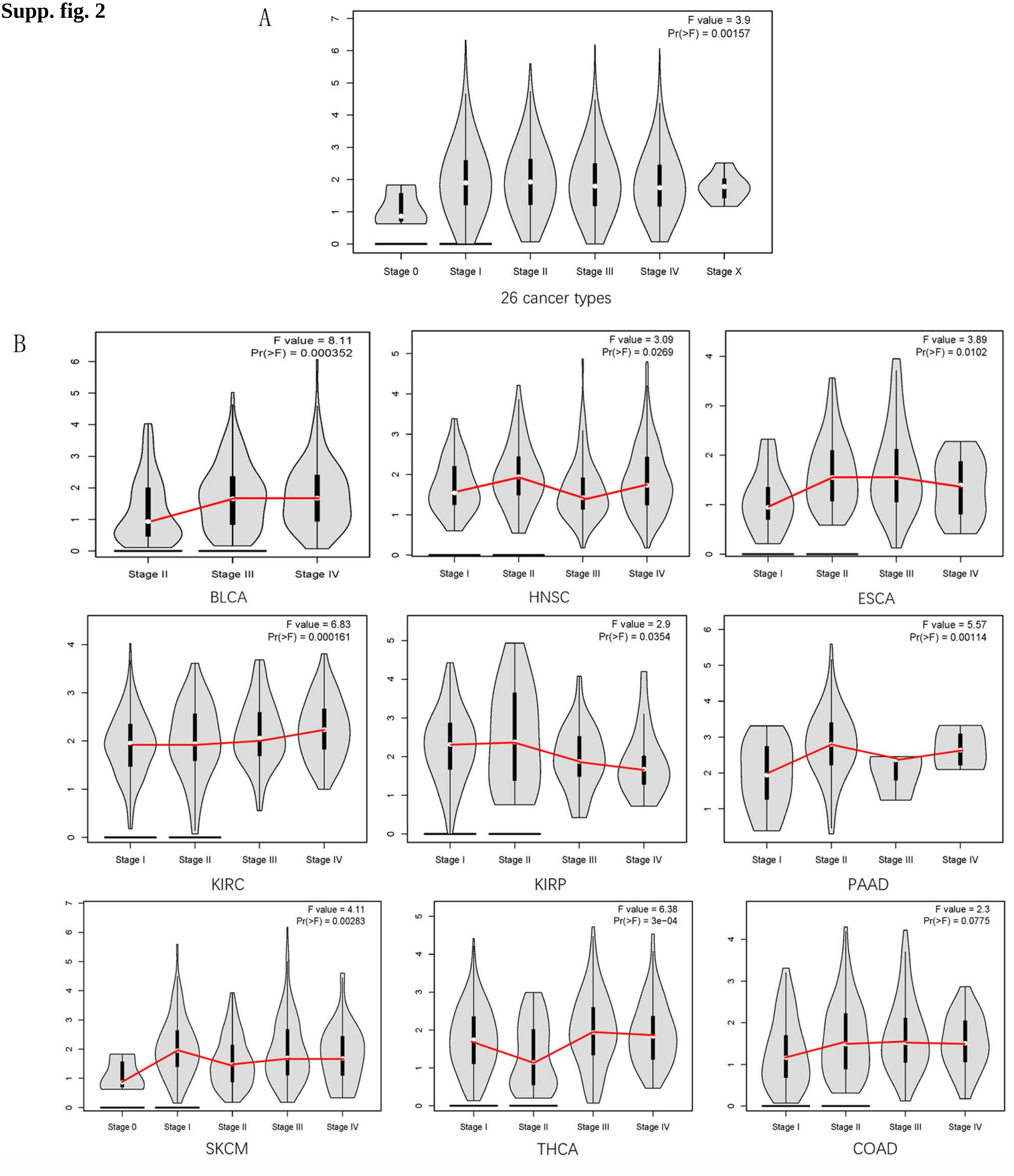
A
F value = 3.9
Pr(>F) = 0.00157
Stage 0
Stage I
Stage II
Stage III
Stage IV
Stage X
26 cancer types
B
F value = 8.11
Pr(>F) = 0.000352
BLCA
F value = 3.09
Pr(>F) = 0.0269
HNSC
F value = 3.89
Pr(>F) = 0.0102
ESCA
F value = 6.83
Pr(>F) = 0.000161
KIRC
F value = 2.9
Pr(>F) = 0.0354
KIRP
F value = 5.57
Pr(>F) = 0.00114
PAAD
F value = 4.11
Pr(>F) = 0.00283
SKCM
F value = 6.38
Pr(>F) = 3e−04
THCA
F value = 2.3
Pr(>F) = 0.0775
COAD

Supp. fig. 3

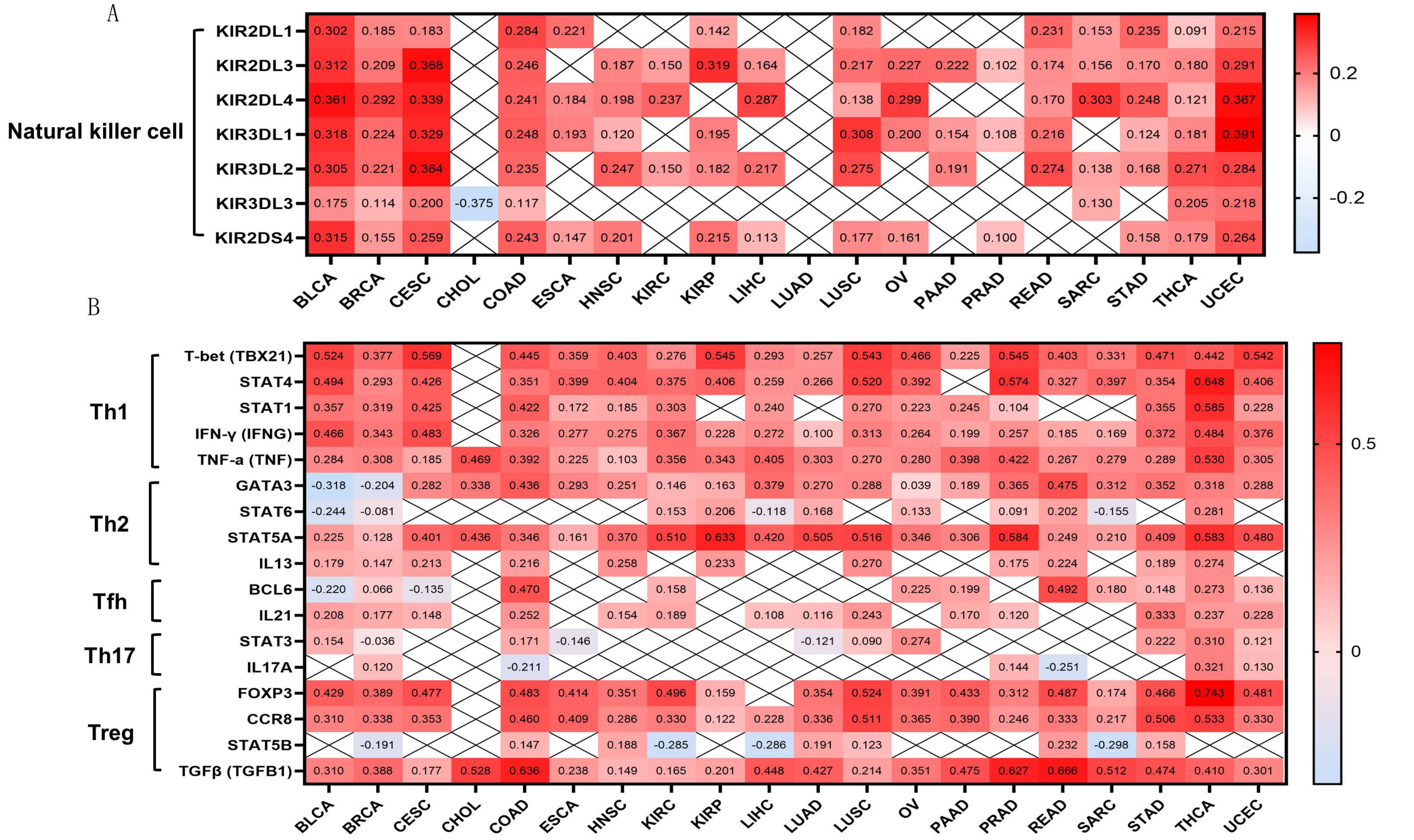
A
Natural killer cell
KIR2DL1
KIR2DL3
KIR2DL4
KIR3DL1
KIR3DL2
KIR3DL3
KIR2DS4
BLCA BRCA CESC CHOL COAD ESCA HNSC KIRC KIRP LIHC LUAD LUSC OV PAAD PRAD READ SARC STAD THCA UCEC
0.2
0
-0.2
B
Th1
T-bet (TBX21)
STAT4
STAT1
IFN-γ (IFNG)
TNF-a (TNF)
Th2
GATA3
STAT6
STAT5A
IL13
Tfh
BCL6
IL21
Th17
STAT3
IL17A
Treg
FOXP3
CCR8
STAT5B
TGFβ (TGFB1)
BLCA BRCA CESC CHOL COAD ESCA HNSC KIRC KIRP LIHC LUAD LUSC OV PAAD PRAD READ SARC STAD THCA UCEC
0.5
0
